# Pre-Clinical Models in Implant Dentistry: Past, Present, Future

**DOI:** 10.3390/biomedicines9111538

**Published:** 2021-10-26

**Authors:** Nicolas Blanc-Sylvestre, Philippe Bouchard, Catherine Chaussain, Claire Bardet

**Affiliations:** 1 Université de Paris, Institut des Maladies Musculo-Squelettiques, Orofacial Pathologies, Imaging and Biotherapies Laboratory URP2496 and FHU-DDS-Net, Dental School, and Plateforme d’Imagerie du Vivant (PIV), 92120 Montrouge, France; nicolasbs.efp@gmail.com (N.B.-S.); philippe.bouchard.perio@gmail.com (P.B.); catherine.chaussain@u-paris.fr (C.C.); 2 AP-HP, Department of Periodontology, Rothschild Hospital, European Postgraduate in Periodontology and Implantology, Université de Paris, 75012 Paris, France; 3 AP-HP, Reference Center for Rare Disorders of the Calcium and Phosphate Metabolism, Dental Medicine Department, Bretonneau Hospital, GHN-Université de Paris, 75018 Paris, France

**Keywords:** pre-clinical research, murine dental implant, human-sized dental implant, osseointegration, biocompatibility, implant models

## Abstract

Biomedical research seeks to generate experimental results for translation to clinical settings. In order to improve the transition from bench to bedside, researchers must draw justifiable conclusions based on data from an appropriate model. Animal testing, as a prerequisite to human clinical exposure, is performed in a range of species, from laboratory mice to larger animals (such as dogs or non-human primates). Minipigs appear to be the animal of choice for studying bone surgery around intraoral dental implants. Dog models, well-known in the field of dental implant research, tend now to be used for studies conducted under compromised oral conditions (biofilm). Regarding small animal models, research studies mostly use rodents, with interest in rabbit models declining. Mouse models remain a reference for genetic studies. On the other hand, over the last decade, scientific advances and government guidelines have led to the replacement, reduction, and refinement of the use of all animal models in dental implant research. In new development strategies, some in vivo experiments are being progressively replaced by in vitro or biomaterial approaches. In this review, we summarize the key information on the animal models currently available for dental implant research and highlight (i) the pros and cons of each type, (ii) new levels of decisional procedures regarding study objectives, and (iii) the outlook for animal research, discussing possible non-animal options.

## 1. Introduction

In the field of dental implant research, experiments have been mainly limited to in vivo studies in so far as translational studies are a prerequisite for any clinical research. Identification of the reasons for the failure and success of dental implant treatments remains the most frequent question in clinical practice. By mimicking the biological condition of an implant, pre-clinical research makes it possible to investigate aspects of peri-implant tissue healing and peri-implant disease development [1].

**Figure 1:**
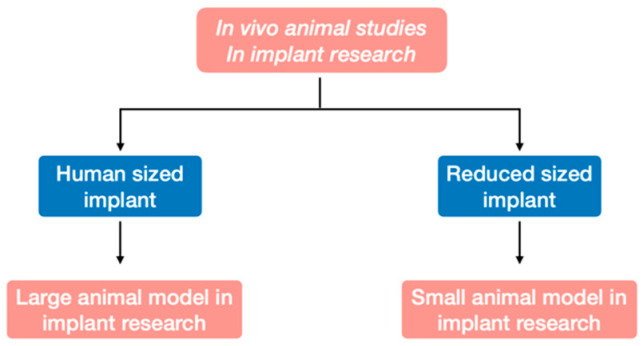
Conventional decision tree for animal model selection in dental implant research: large vs. small animal models.

Traditionally, two types of in vivo implant studies have been conducted depending on the implant size used: (1) experiments in large animal models (dogs, pigs, and non-human primates [NHPs]) for studying human-sized implants and (2) experiments in small animal models (rabbits, mice, and rats) for studying adapted “implants” (Figure 1). Nonetheless, taken separately, no animal model is able to shed light on all three levels of implant osseointegration (macro/micro/nano). For example, assessment of implant occlusion (macro level) is limited to large animal models, but these models do not allow the analysis of molecular interaction at the bone/implant interface (micro level). Moreover, limiting the selection criteria to animal size amounts to ignoring other species-specific characteristics (anatomy, physiology, etc.) of each model. Overall, animal model justification in implant studies is complex and requires decision support tools.

Since the 1950s, researchers have been widely encouraged to find alternatives to animal testing and improve animal welfare and research quality. In many countries, the principles of Replacement, Reduction, and Refinement (the “3Rs”) are now embedded in national and international legislation, redefining the use of animals in scientific procedures with the establishment of the ARRIVE (Animal Research: Reporting of In Vivo Experiments) guidelines [1,2,3]. Nowadays, in vitro studies play a leading role in the development of tissue bioprinting, organoids, or organ-on-a-chip, which have emerged as promising approaches for replacing animal experiments in basic research. Guided by the principles of the 3Rs, trends in use as a function of this size-based classification have changed, with large animal models mainly being used for clinical studies, small animal models employed preferentially for pathophysiological pathway analysis [4], and the substantial development of in vitro methods. 

The present review aims to clarify these trends in the use of animal models in dental implant research and highlights the pros and cons of each of these models. It also discusses the outlook for animal research and emerging decisional procedures regarding study objectives, as well as currently available and promising non-animal options. 

## 2. Large Animal Models in Implantology

### 2.1. Non-Human Primate Models

Many species of NHPs have been used as bone disease models due to similarities of their physiology to that of humans. NHPs have considerable genetic homology with humans, which allows the use of numerous human probes for genetic studies [5]. Further, they develop similar bone diseases to humans, such as osteoporosis and age-related bone loss [6,7].

#### Pros and Cons of the Models

**Figure 2:**
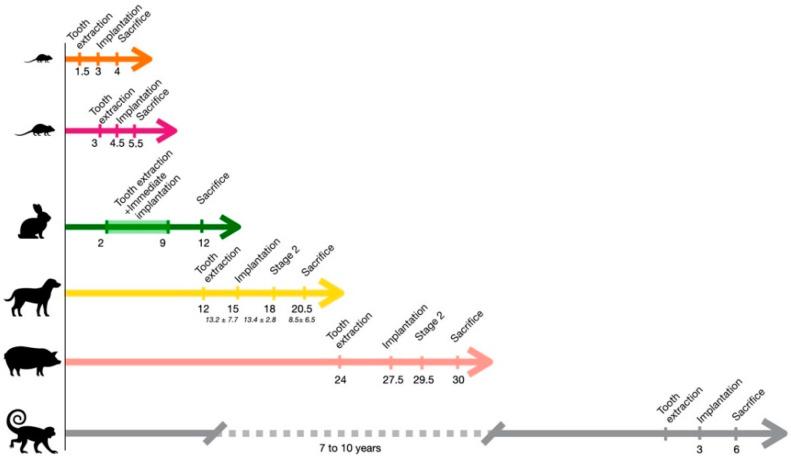
Representative duration of dental implant protocols by type of animal model. Time is expressed in months (unless indicated otherwise) from birth to sacrifice (values for large animals adapted from Schwarz et al. [9]).

NHPs were naturally chosen for dental procedures for their dental similarities with two dentitions (deciduous and permanent teeth). Even if periodontitis does not often occur naturally, plaque accumulation may occur, potentially progressing to gingival inflammation [8]. They are therefore one of the best models for oral procedures, including dental implant surgery. On the other hand, these similarities are also considered a disadvantage in fundamental research where procedures tend to be as short as possible, protocol duration in NHPs being the longest compared to all other models (Figure 2, Appendix A.1).

**Figure 3:**
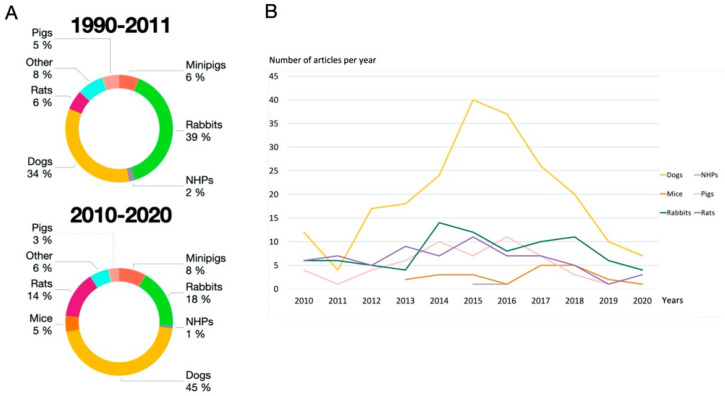
Distribution of animal model studies in implant research (**A**) from 1990 to 2011 (adapted from Stadlinger et al. [2]) and from 2010 to 2020 (NHP: non-human primate); (**B**) distribution of publications over the past 10 years.

**Figure 4:**
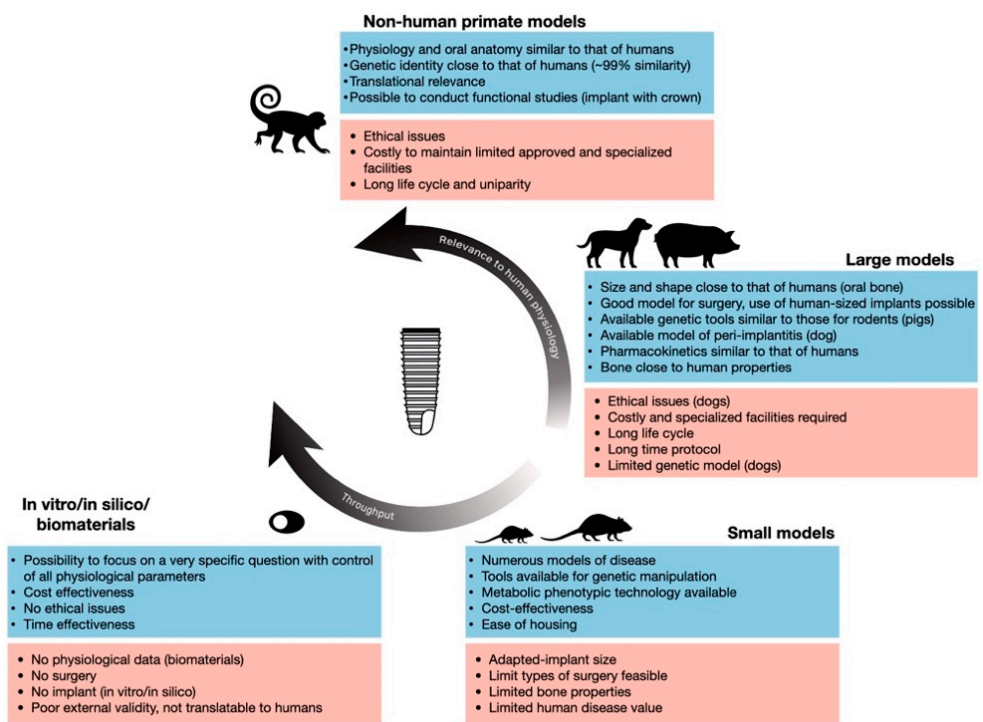
Key advantages and disadvantages of the different types of models used in dental implantology research (adapted from Kleinert et al. [11]).

Furthermore, for ethical reasons, in addition to costs and housing difficulties, NHPs have almost completely stopped being used [10] in accordance with international legislation, except for the assessment of major innovations or new treatments already validated in another large animal model. Consequently, the number of research studies using NHPs has decreased (Figure 3A) with few articles on dental implant procedures, most published before 2015 (Figure 3B). Pros and cons are summarized in Figure 4.

### 2.2. Canine Models

Before the development of implantology and in contrast to NHPs, dogs were considered the natural model for periodontitis. Indeed, most canines can develop periodontal disease with a conventional transition process from gingivitis to periodontitis [12,13]. This natural periodontitis reproduces human periodontitis from both the microbiological [14] and clinical [10] points of view. Clinically, this model makes it possible to investigate commonly used grading criteria: pocket depth with marginal alveolar bone loss and marginal recessions [10]. Periodontitis severity normally decreases from the first premolar to the first molar [15]. 

#### Pros and Cons of the Models

A recent report published by the National Association for Biomedical Research based on the US Food and Drug Administration data has shown that dogs were key in developing 22 out of the 25 drugs most prescribed in the US in 2014 [16,17]. In implantology, successful pre-clinical study designs in dogs have been used to test general approaches and regenerative therapies, such as the use of growth factors and barrier membranes [18]. Advantages of dog models include the ease of management and manipulation before surgery and during postoperative oral hygiene procedures and reducing bias between animals; however, they have the disadvantage of dogs being companion animals with the associated ethical implications [19]. As the use of these models was well established in the periodontal research community, they were naturally transposed to the implantology field [20,21] and validated in peri-implantitis models [22,23,24]. Such natural periodontal lesions appear after several years which is a disadvantage, but they are usually accelerated with a soft diet and submarginal ligatures [9].

Thanks to this extensive history of use in basic research over more than 40 years, canine models have gained prominence and are now widely used in implantology, having been employed in 215 out of 479 studies reported this last decade (Table A2). Dogs are considered large animal models in implantology and the use of human-sized implants is common [4]. Bones are similar to those of humans in terms of water, organic, volatile inorganic, and ash fractions [25]. In addition, dogs have a mixed microstructure bone with secondary osteons mainly in the center of the cortical bone, with plexiform organization on each side [26]. Plexiform bone is characterized by a rapid bone apposition process. In humans, this organization is only found in children during rapid growth to improve mechanical strength against fracture. 

There are, however, notable differences in terms of weight and size between dog breeds, in some cases, increasing discrepancies with human bones [19]. Furthermore, the rate of trabecular bone remodeling differs between humans and dogs, and also between bones in the same animal (with bone turnover rates from 12% for the talus to nearly 200% for the lumbar vertebral body [19,27]). Similar differences have been observed in cortical bone [19,28] and between oral bones, with a bone remodeling rate two-fold higher in the mandible than the maxilla [29]. Aside from age, which affects bone turnover and response to implants [30], this specificity has to be considered for determining the implantation site.

Experiments are usually performed in 1-year-old individuals with full adult dentition but can be performed in dogs up to 2 years of age [31] (Figure 2, Appendix A.2, Table A1). Pros and cons are summarized in Figure 4.

### 2.3. Swine Models

Swine, both pigs and minipigs, is one of the main species used in translational research. Pigs have the advantage of anatomical, physiological, metabolic, and genetic similarities to humans. Bone studies were mostly conducted on porcine models in the 1970s for studies on infectious bone diseases, [32] surgery training and toxicology testing, [33] and researchers rapidly extended this model to the field of implantology in the 1990s [34]. Their use in biomedical research has been growing considerably in recent years as it has come to be considered an optimal model for many human diseases. Pigs are now used in various fields of biomedical research, including genetics [35] and clinical research (e.g., organ transplantation and cancer [36]). 

#### Pros and Cons of the Models

The use of pig models is justified in dental implantology by the similarities of the periodontium to that of humans both anatomically and physiologically [37]. Indeed, pig bone has a similar Haversian structure to that of humans [38] and also a similar bone mineral density, [25] with minimal differences in minimum diameter and number of lacunae per osteon [39], and bone remodeling rate (1.2–1.5 mL/day in pigs vs. 1.0–1.5 mL/day in humans) [19]. Nonetheless, there are some differences: notably, pigs have a denser trabecular network and a higher bone mass, [40] and the maximum diameter, perimeter, area, and circularity of the osteons also differ [39].

Generally, from a research point of view, commercial breeds of pig, or farm pigs, have multiple disadvantages. First, the development of pigs results in rapid growth rates and excessive bone weight which is a disadvantage compared to other species [6]. Secondly, pigs tend to be difficult to handle due to their potentially aggressive temperament, heavy weight (up to 350 kg in the case of an adult domestic pig [5]) and high housing costs. Any repetitive procedure, such as oral hygiene maintenance, cannot be carried out without trained technicians [41]. Further, it is difficult to train pigs and postoperative healing may be jeopardized if they can access materials to chew (e.g., metal grid bars) [42]. The breeding of minipigs has resolved some of these issues [43] and has considerably helped to widen the use of pig models.

Minipig models are nowadays a standard tool for dentistry research [4]. There are almost 50 breeds of minipigs available worldwide [44]. As adults, depending on the breed, mini-pigs weigh between only 35 kg (Göttingen breed) and 95 kg (Hanford breed) [45], facilitating their housing. They reach sexual maturity early, at 4 to 6 months of age (Table A1). As they result from selective breeding, they are not considered transgenic or genetically modified animals [46] and their physiology and anatomy are not far different from those of conventional pigs [33]. In implantology, both pigs and minipigs show anatomic characteristics close to those of humans, allowing the placement of commonly used dental implants (6–10 mm in length/3 to 4.8 mm in diameter) which is a tremendous advantage compared to small animal models [4]. Another major advantage of minipigs is the ability to perform long follow-up with multiple surgical steps [47] and age-related studies [48]. On the other hand, to avoid the costs of breeding, animals are usually ordered from commercial laboratories for each experiment. 

The duration of a conventional protocol differs between studies and between pig models (farm pigs versus minipigs) (see Appendix A.3). Pros and cons are summarized in Figure 4.

### 2.4. Other Large Animal Models

Other large animal models have not been widely used in implantology (Table A2). The ovine long-bone model has been mainly used for research on surgical techniques (vertical ridge augmentation [49], drilling procedures [50,51,52]), and implant surface properties [53]. Overall, in sheep, there have been relatively few studies with differing protocols and objectives. Only one study has been conducted in the mandibular bone to assess the effect of implant coating on a titanium implant [54]. Other large animal models have been tested, such as the sika deer for their antlers [55], and the goat for surgical experimentation [56] and implant osseointegration (mainly in the pelvic region) [57,58].

### 2.5. Conclusion on the Use of Large Animal Models in Dental Implant Research

**Figure 5:**
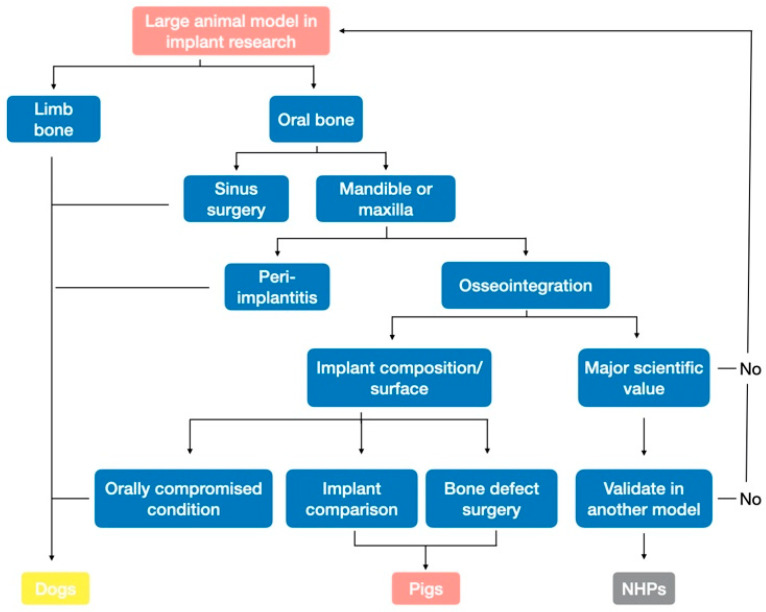
Large animal model selection in dental implant research (NHP: non-human primate).

Nowadays, within the binary “large vs. small animal models” classification (Figure 1), a new level of decisional procedures has emerged regarding study objectives, with the establishment of sub-classes according to species-specific characteristics of each large animal model. Pre-clinical surgical procedures (e.g., sinus or bone augmentation) with human-sized implants on large animals can be categorized (Figure 5):

- NHPs are no longer used in Europe and are only used elsewhere in already accredited procedures. NHP models, particularly the baboon, should be considered a confirmation model reserved for studies on major advances providing substantial added scientific value, already validated in another model.
- Pigs and minipigs are the new pioneers, having replaced dogs in procedures. The minipig appears to be an ideal model for studies of bone regeneration around dental implants when placed at intraoral sites.
- Dogs should only be used when pigs cannot be used to address the question of interest (mainly for compromised oral conditions, sinus surgery, and peri-implantitis procedures). In particular, dog models should be preferentially employed for studies conducted under compromised oral conditions (biofilm).

## 3. Small Animal Models in Implantology

### 3.1. Rabbit Models

The popularity of these models stems from the work of Sawin et al. in orthopedics in the 1940s, supported by descriptions of rabbit breeding, anatomy, and surgical protocols [59,60]. At the end of the last century, approximately 35% of musculoskeletal research studies were performed using rabbit models [61]. This widespread documentation from biomedical research led to the use of this animal for in-vivo bone studies. In 1997, Mori et al. used a rabbit model to improve our understanding of the physiological process of osseointegration in rabbits with induced osteoporosis [62]. Since then, rabbits have been used intensively, representing 27% of dental implant research in animals [1]. Interestingly, however, this figure has fallen sharply with just 86 studies (11%) over the last decade (Figure 3B), reflecting the overall decrease in their use in research [63].

#### Pros and Cons of the Models

According to the American Rabbit Breeders Association, there are 49 rabbit breeds [64]. Of the various breeds, the New Zealand White rabbit (5 to 6 Kg) is the most commonly chosen for implantology research (Table A1). This species is of particular interest with the advent of transgenic rabbits (for hormone regulation [65], diabetes [66], and osteoporosis [67]). Thanks to its accelerated skeletal maturity (at around 6 months of age) and rapid bone turnover (faster than that in primates), the rabbit is a convenient model for laboratory research [68]. On the other hand, this rapid turnover could introduce a bias in long-term studies, making results difficult to interpret with respect to human biology [19]. Furthermore, the skeleton of the mature rabbit is fragile, representing only about 8% of its body weight [69]. Histological analysis of compact bone has shown rabbit bone to be one of the most different from humans, with major differences not only in Haversian canals and secondary osteons but also in vascularization [70,71].

Surgical protocols to study osseointegration have been developed in two main areas in rabbits: (i) extra-oral models in long bones (femur and/or tibia) and (ii) oral models (mandible, maxilla, and sinus) (see Appendix A.4). Pros and cons are summarized in Figure 4.

### 3.2. Rat Models

Rats are a good starting point for testing new procedures thanks to the ease of housing and relatively low costs, compared to those of large animal models, as well as the extensive history of their use in scientific experiments [25]. Rat physiology, especially in bone tissue, suggests it would be useful for research in certain areas. The growing rat is a well-known model for evaluating the effects of endocrine, nutritional and environmental factors on peak bone mass but is not appropriate for adult human skeleton studies due to the presence of cellular pathways not present in human adults [72]. Bone mass gain, in parallel with the long bone elongation, mainly occurs during the first 6 months of life [72], though some authors consider that the long bone grows continuously for at least 1 year with a gradual transition from modeling to remodeling with age [73], a transition that does not occur uniformly across bones [74]. Due to this longitudinal bone growth, a margin of at least 1 mm from the growth plate of the tibia should be left intact if experimentation starts around 10 months of age, an issue to be considered in dental implant studies [74]. Sex and hormones are also key parameters in rat research. At 8 months of age, males were found to have 22% greater bone width and 33% greater breaking strength than females in the tibia [75]. The role of hormones has been put to good use in an ovariectomy model. Rats, as for any rodents, do not have natural menopause, but the ablation of the gonad is a good model for artificial menopause [76] and therefore for the analysis of osseointegration in a model of pathological bone.

#### Pros and Cons of the Models

For implantology purposes, the size of the rat is a mixed blessing. The lower costs and ease of housing and handling favor the use of this animal. On the other hand, only small human implants can be used, and most of the time, implants need to be adapted.

Two rat breeds are commonly used: (1) Wistar rats (from the Wistar Institute) are one of the oldest and still considered one of the best rat models. By adulthood, they reach 500 g [77] which places them in the mid-upper range of small laboratory animals. (2) Sprague Dawley rats, developed from Wistar rats, have an adult weight of up to 300 g [5] and are one of the breeds most widely used in pre-clinical studies [78]. They have been used as a model for osteoporosis, and for analyzing the effects of calcium supplementation on bones [79] (Table A1). Various protocols have been developed in rats depending on the implantation site (see Appendix A.5). The number of research studies using rats has increased during the last decade (Figure 3A) and confirms the scientific interest in this model [80]. Pros and cons are summarized in Figure 4.

### 3.3. Mouse Models

The mouse is the animal most commonly used in laboratory research. It was the first laboratory animal model established for genetic- and aged-related changes in bone [81] and used for full genomic analysis.

#### Pros and Cons of the Models

Among all mouse strains, C57BL/6 is the most commonly used, almost 20,000 papers having been published referring to research using this strain in 2019 [82]. Within the same strain, different sub-strains show notable genetic and phenotypic differences [83]. It is therefore important to determine, when planning a research study, which type of animal is needed.

From a bone point of view, mice have similar growth characteristics to rats, with even more marked size-related advantages and disadvantages. It is therefore inappropriate to carry out an implant study in mice if the same model has been developed in rats. Nonetheless, mice have some characteristics which distinguish them from other laboratory animals.

The main advantage they offer over other small animal models is the existence of numerous knockout and transgenic mice. This factor is even more important with the emergence of new tools to develop genetically engineered mouse models. Transfection or viral vector transduction are routinely applied methods for random DNA integration [84], while the CRISPR-cas9 system for gene editing [85] is an emerging technology that extends the scope of research in this field [86].

In implant studies, this model has been used for a long time but primarily for extra-oral approaches due to technical and surgical complications [4], the reason most often mentioned being the difficulty of access due to the mouth size and range of opening of mice. Some authors opted to develop a more accurate model by working on the mouse maxilla [87]. A limitation of this model is the limited cortical bone remodeling and the lack of the Haversian structure in cortical bones. Indeed, rodent long bones are mainly composed of primary bone and a minimal proportion of cancellous bone [6]. The counterpart of this biological issue is the small amount of cancellous bone site for implantation studies [88].

As for rats, protocols are markedly heterogeneous, and it has not been possible to establish a gold standard (See Appendix A.6). Pros and cons are summarized in Figure 4.

### 3.4. Conclusion on the Use of Small Animal Models in Dental Implant Research

**Figure 6:**
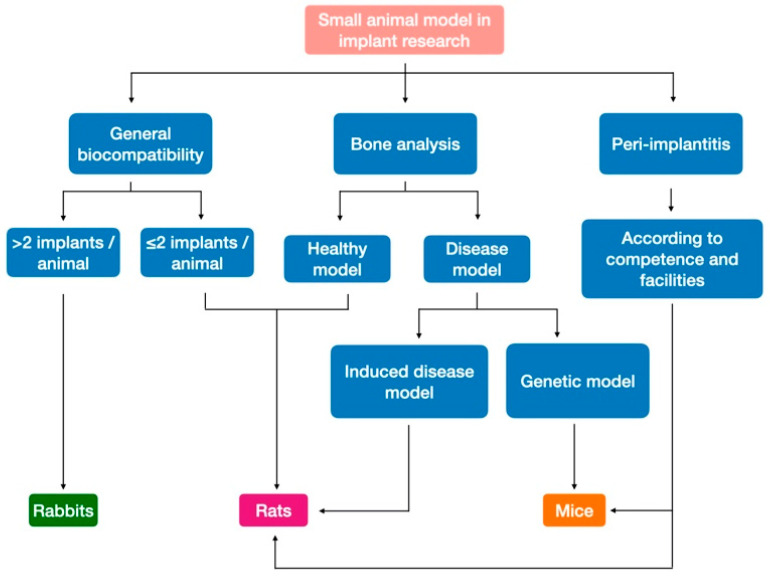
Small animal model selection in dental implant research.

Smaller-sized implant models and biocompatibility studies should be performed on small animal models as cell toxicity does not require implant-shaped material (Figure 6).

- Rabbits should be recommended for biocompatibility studies if large numbers of implants are needed per animal, their availability in large numbers appearing to be the only advantage of this model.
- Other questions should be addressed using rats, which are suitable for biocompatibility and common bone analysis in healthy models.
- Mice are still the best option for human disease models with the existence of numerous knockout and transgenic mice models. Peri-implantitis procedures are also an emerging field in this species.

## 4. Future Challenges and Strategies

### 4.1. The Outlook for Animal Models

The framework of the 3Rs re-defined animal model applications in implantology by completely rethinking our way of operating Although in vivo studies remain essential to investigate specific challenges in implantology, in vitro approaches play a leading role in developing protocols.

**Figure 7:**
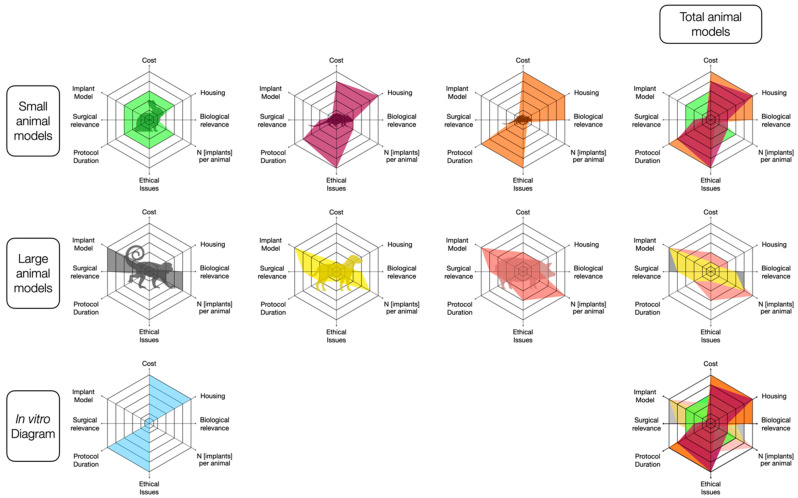
Overall appeal assessed by animal model. Diagram based on key elements of interest in pre-clinical research: cost, housing and husbandry requirements, protocol duration, biological relevance, N [implants] per animal, ethical issues, surgical relevance, and resemblance to the human implant model. For each criterion, the models were ranked (from best [1, outer line] to worst [6, inner line] (Table A3). Overlaying diagrams by animal size also shows that small and large models have very similar characteristics to others in the same size group. The diagrams indicate that the in vitro/in silico and biomaterials study type was ranked first for four criteria (cost, housing and husbandry requirements, ethical issues, and protocol duration) and last for two criteria (surgical relevance and implant model), as they are not addressed by such studies, while two criteria were not applicable to this last category (biological relevance and number of implants per animal). Comparison with the global diagram shows that the mouse model diagram is the closest to that of the in vitro/in silico/biomaterial models.

Overlaying diagrams by model size (small or large) shows that models within each category offer similar characteristics. Interestingly, rating comparable criteria for each model, (i) although mice are small, the mouse model seems to have numerous advantages for implant studies and (ii) in vitro/in silico models and analyses of biomaterial properties are ranked top, highlighting their great potential in the field of dental implantology (Figure 7). 

### 4.2. Development of Replacement Strategies

The principles of the 3Rs were developed over 50 years ago, aiming to encourage alternatives to animal testing and improve animal welfare and research quality where the use of animals could not be avoided.

#### 4.2.1. In vitro Biocompatibility and Cytotoxicity Analyses

In vitro human or animal cell-based studies on modified surfaces for dental implants allow the assessment of toxicity and characterization of osteoblast adhesion to the implant, or the impact of any added processing steps on the implant surface [89,90]. For example, when a new nanoparticle treatment is developed, in vitro studies are needed to test implant treatment viability during cell interaction [91]. Similar studies are needed to test bioactive [92,93,94] or peptide (e.g., RGD [95]) coatings, or the incorporation of antibiotics [96] or growth factors (e.g., bone morphogenetic proteins [97]).

In vitro studies can also assess the impact of given clinical methods on a device [98]. By applying a procedure directly to the implant, for example, studying the effect of various polishing methods on bacterial colonization [99,100,101], the need for an animal model can be reduced and it may be easier to focus on the interaction of interest. As titanium is the main material used in dental implant surgery, experiments have mainly focused on titanium powder or titanium disks. For example, in vitro studies showed that some surfaces induce the generation of toxic particles, certain surfaces being more toxic than others to oral epithelial cells [102]. Other materials, such as zirconium implants, have also been tested for cell biocompatibility and mechanical properties [103]. 

#### 4.2.2. In Vitro Models of Response to Implant and Associated Biofilm

For more biological issues, in vitro models were developed at the end of the 1990s to assess the possibility of answering simple questions without the use of animals [104,105]. According to Mombelli et al., in vitro models were relevant for studying: (i) the reaction of micro-organisms to the presence of implants, (ii) the reaction of implant-associated micro-organisms to antimicrobial agents, and finally, (iii) the reaction of the host tissues to the presence of implants contaminated with micro-organisms [105,106]. In vitro studies have subsequently been applied to the issues of hypersensitivity, and immune and pro-inflammatory responses [107]. Biofilm assays are also a common in vitro procedure for the analysis of antibiotic resistance. Adapted to implantology, complete implants or titanium chips are used in cell culture in the presence of bacteria, allowing the analysis of bacteria adhesion and biofilm construction. Once the biofilm is stable, chlorhexidine [108] or antibiotics can be added to the medium to observe their performance. In the same process, infected implants can be placed in contact with animal or human cells to study their interaction [106]. 

#### 4.2.3. In Vitro Physical and Mechanical Evaluation

Once biocompatibility and cytotoxicity have been demonstrated, protocols for physical and mechanical testing can assess the resilience to different loads in pre-load models, monitoring numerous variables related to the implant–abutment connection [109], such as force used for screw tightening [110], ability to withstand a long-term load (assessed by direct strength testing) [111], and chewing cycle (simulated by an artificial mouth) [112,113]. Further, esthetic comparisons can be made in terms of abutment titanium visibility [114].

Finally, in vitro rather than animal models should be used to study the improvement and/or development of future technologies, such as computer-guided navigation for implant placement [115], laser procedures [116], or comparisons between different scanning methods [117].

**Figure 8:**
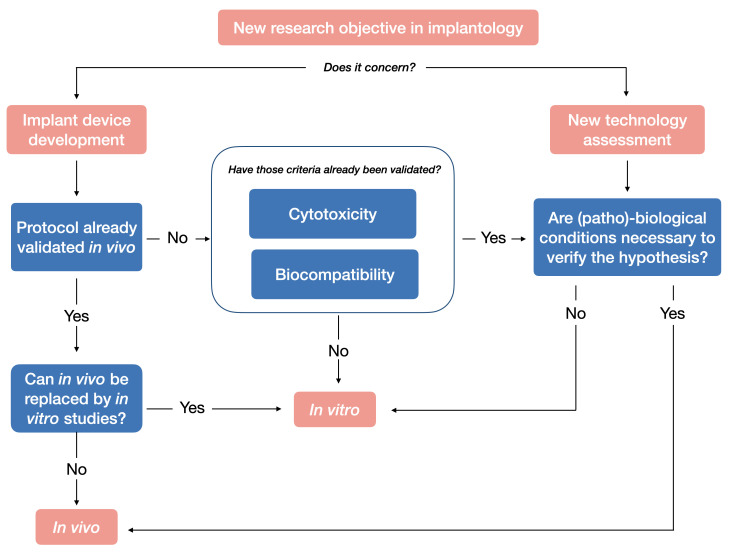
Research protocol selection in dental implant research: in vivo vs. in vitro.

The 3Rs are now widely embedded in national and international legislation and regulations on the use of animals in scientific procedures. Following these principles, before conducting an in vivo study, it has to be considered whether it is possible to replace the use of animals with alternative methods. Indeed, in implantology, before any in vivo test, in vitro analysis is essential for implant development. Before choosing which animal is needed for a procedure, the first decision is whether we can avoid using animals at all (Figure 8).

## 5. Conclusions

Biological advances in large animals have narrowed the gap between large and small animal model applications, as it is now possible to perform genetic analysis in dogs while it was previously only possible in small animal models, and at the same time, technological advances have enabled reductions in instrument size, and therefore the manufacturing of small implants is compatible with mouse size. The distinctions between these two groups are small, but the specificities of dental implant models allow rational decisions concerning their use to maximize scientific impact and benefits. 

Last but not least, nowadays any decision-making process dealing with animal sacrifice in research raises the key question of its scientific necessity, especially in the development of dental implant protocols dealing with elective surgeries (Figure 4). Considerable efforts have been recently made to replace animal studies with in vitro studies, which enable mechanical and physical characterization of dental implants. Thus, when the question of the use of animals in implant surgery research nowadays arises, another question must always follow: “can we do otherwise?”.

## Appendix A

### Appendix A.1. Research in Non-Human Primates

In 2010, the European Parliament issued a directive which is still in force: “The use of non-human primates should be permitted only in those biomedical areas essential for the benefit of human beings, for which no other alternative replacement methods are yet available” [118].

Furthermore, there are risks associated with handling due to the possibility of zoonotic disease transmission [41,119] but also biological and behavioral responses due to stressors such as separation from their familial environment [120].

The duration of a conventional protocol is around 6 to 9 months with a first healing time of 3 months after tooth extraction and 3 to 6 months after implantation (Figure 2). Protocols are usually performed on adult animals, from 7 to 10 years old, this allowing the use of human-sized implants (Figure 5). Laboratory breeding and reproduction are therefore not feasible, and animals are acquired for the protocol. Such studies have investigated the healing process after sinus floor elevation [121,122,123], improvements of analysis techniques [124], and clinical questions concerning soft-tissue response around combined tooth–implant-supported prostheses [125,126]. Old World monkeys such as baboons, mandrills, and macaques are preferred, as their long bones have a dense Haversian structure, with thin layers of endosteal and periosteal bone [127]. For anatomical reasons, the use of Rhesus macaques has to be avoided, their adult size and weight (6.5 to 12 kg vs. 21.5 kg for male baboon) [5] being too small to be considered a “large animal model”.

Summary: In accordance with international legislation, NHPs should no longer be used except for the assessment of major innovations or new treatments already validated in another large animal model (Figure 5).

### Appendix A.2. Research in Canine Models

The most commonly used dog breed is the beagle due to the ease of care. Its mean adult weight is around 16 kg [5] allowing the use of human-sized implants. For this type of study, all animals are acquired from accredited laboratories. Mainly carried out in long bones in the past, protocols have now been developed for oral bone models which have more appropriate characteristics for the analysis of osseointegration. Similarly, peri-implantitis has been successfully transposed to oral implant studies, facilitating the use of oral bone models (Table A1).

The duration of a conventional protocol is slightly shorter than in NHPs, with a first phase of 3 months after tooth extraction and 2 to 3 months of implant healing (Figure 2).

**Table A1:** Summary of species characteristics and use in implant protocols.

	Non-Human Primates	Pigs	Canines	Rabbits	Rats	Mice
Species most frequently used	Baboon, mandrill and macaques	Pigs: Domestic pigs Minipigs: Hanford of Göttingen breed	Beagle	New Zealand White rabbit	Wistar rats, Sprague Dawley rats	C57 Black/6
Age of use	7 to 10 years old	2 to 3 years old	1 to 2 years old	6 to 9 months old	2 to 3 months old	8 weeks old
Protocol duration	6 to 9 months	12 months	5 months	2 to 4 weeks (long bone) Up to 3 months (oral bone)	2 to 6 weeks (long bone) 2.5 months(oral bone)	4 weeks (long bone) 2 to 3 months (oral bone)
Weight	21.5 kg	Pig: 350 kg Mini-Pig: 35 to 95 kg	15 kg	5 to 6 kg	Sprague dawley: 70 to 300 g Wistar rats: up to 500 g	30 g
Implant size	Human-sized	Human-sized	Human-sized	Human-sized Adapted implant	Adapted implant: 1.5 mm diameter, 2.5 mm length	Adapted implant: 1 mm diameter, 2 to 3 mm length (long bone) 0.6 mm diameter, 2 mm length (maxilla)
Trend	Falling into disuse	Any study related to implant surgery under healthy conditions	Peri-implantitis, sinus and genetic studies	Falling into disuse	Systemic conditions (diabetes, hormones), poor bone quality models, ease of breed and use	Genetic studies, knock-out protocols, peri-implantitis

#### Appendix A.2.1. Long Bone Models

To evaluate implant osseointegration, dental implants were placed in dog leg bones, but this is rare nowadays, less than 20 studies having been reported over the past 10 years (Table A2).

Despite an obvious bias of studying implants loaded on a quadrupedal gait model, protocols involving implant placement on limbs allow the use of a large number of implants, thus reducing the sample size (in one study, up to 75 implants having been tested in the radius of just 6 dogs [128]). Though front limbs can also be used, [128] most of the time both tibias are used, as they offer a large quantity of bone. For this kind of study, implants 3.75 mm in diameter and 10 mm in length are the most common, two to three implants being used per tibia [129]. The large amount of bone available allows the creation of surgically created defects to analyze bone regeneration associated with dental implants. Properties of membranes [129] or new grafting compounds [130] have been tested. New implant devices such as implant extenders have also been tested before clinical use [131]. The proximal tibia is commonly used for drilling protocols, to test the impact of drilling in early stages of osseointegration and implant stability [132,133,134,135,136,137], different implant surfaces [138,139,140,141,142], and biomechanical properties (insertion torque [133], response to compressive stress [143]).

**Table A2:** Distribution of implant location by animal and year over the past 10 years based on research articles in MEDLINE, including all experimental implant studies on animals.

		2010	2011	2012	2013	2014	2015	2016	2017	2018	2019	2020	Sum
Canines		12	4	17	18	24	40	37	26	20	10	7	215
	Long bone		1	5	3	2	3	2	1	2			19
	Oral bone	12	3	12	15	22	37	35	25	18	10	7	196
Non-human primates		1		1			1	1					4
	Oral bone	1		1			1	1					4
Mice		1			2	3	3	1	5	5	2	1	23
	Long bone	1			1		1		1	1	1	1	7
	Oral bone				1	2	2	1	4	4	1		15
	Other					1							1
Pigs		4	1	4	6	10	7	11	7	3	1		54
	Long bone						1	1	2				4
	Oral bone	4	1	3	2	9	4	10	2	3			38
	Skull			1	4	1	2		3		1		12
Rabbits		6	6	5	4	14	12	8	10	11	6	4	86
	Long bone	6	6	5	4	11	9	6	10	9	4	3	73
	Oral bone					3	2	2		2	2	1	12
	Skull						1						1
Rats		6	7	5	9	7	11	7	7	5	1	3	68
	Long bone	3	5	4	7	4	9	6	6	4	1	1	50
	Oral bone	3	2		2	1	2	1	1	1		2	15
	Skull			1		2							3
Other		3	2		2	5	3	4	2	6	1		28
	Long bone	2	1		1	2	2	2	1	1			12
	Oral bone					3		2	1	2			8
	Other	1	1		1		1			3	1		8
Sum		33	20	32	41	63	77	69	57	50	21	15	478

#### Appendix A.2.2. Oral Bone Models

Thanks to anatomical similarities, oral bones have been intensively used for research on surgical techniques (Table A2).

- Studies in the maxilla: Rehabilitation of the posterior area is still challenging in clinical practice. Due to sinus pneumatization, the use of small implants versus sinus augmentation is a routine clinical question. The main advantage of the maxillary bone in dogs is the possibility to perform sinus grafting or sinus augmentation procedures. The model is now well established [144] and provides data on, for example, the effect of different depth implant penetration [145], utility of bone grafts [146], and the effect of new materials such as platelet-rich fibrin [147] which inform clinical decision making. Guided bone regeneration substitutes have been tested for augmentation at peri-implant defects to assess the biocompatibility and efficiency of new materials [148], membranes [149], and different implant compositions [150]. The anterior area has also been used to test ridge expansion. This type of surgery can be followed by vertical and horizontal resorption of the bony wall. As histological measurements are not possible in humans for ethical reasons, the performance of such techniques in the dog maxilla has made it possible to investigate the healing process and bone remodeling [151,152].
- Studies in the mandible: Healing patterns of the mandible, both of the bone [31,153] and soft tissue compartment, are now well characterized [154,155,156,157]. As a result, new techniques have been developed to standardize or even to automate [158] osseointegration analysis. New robotization tools have been developed for biomechanical testing in parallel with 3D modeling [159]. Combined technologies, like the overlaying of micro-computed tomography and STL images of an implant, have been developed to analyze hard and soft tissue volume [160].
- Successfully applied to the mandible, conventional protocols have provided clues to answering other clinical questions concerning issues such as the importance of the vertical position [161,162,163], the implant–crown ratio [164], and implantation in residual roots [165]. Drilling protocols with new techniques [166], sizes [167] or speeds [168] have been analyzed. New surgical methods, like the socket-shield technique [169,170], bone-ring technique [171,172], flapless protocols, and ridge augmentation have improved our understanding of peri-implant tissue healing. The influence of immediate/delayed implant placement on the peri-implant bone [173] and soft-tissue [174] formation has been well documented [175,176]. Post-extraction socket healing, with or without implants [177], has been tested, allowing the basic protocol to be modified to prevent dehiscence [178] or manage the jumping distance between implant and vestibular bone [179]. Bone response to biomechanical loading over time [180,181] or compressive stress [143], excessive loading [182], or lateral force [183] has been studied.
- Biomaterials is a major field of implant research in dogs, especially for tissue augmentation with membranes [184,185,186], xenografts (DBBM [187,188,189,190]), allografts [191], or alloplastics [18,192], but also biotherapeutic proteins (rhBMP-2) [193,194,195,196], progenitor cells [197], and stem-cells [198,199], and the use of platelet-rich fibrin [200,201,202].
- Studies in the mandible have also allowed comparisons between implants. The mandible is large enough to test different implant systems [203], as well as implants with different shapes [204,205], lengths [206], surfaces, and grooves [207,208,209]. The race to find the best alloy, or surface finish, is still open. New materials like zirconium [210], PEEK [211], tentalum [212], and titanium alloys [213] have also been used to enhance osseointegration. Implant surface properties is a field that attracts the attention of many researchers. Old techniques have been improved with the addition of molecules like magnesium [214], plasma projection [215], or chemical treatment [216] and new techniques have been developed with nanocoatings [217] or biofunctionalization [218]. Comparisons have been made between implants with differing abutment shape [219,220], composition [221,222], or microstructure, [223] and different protocols, e.g., platform switching [224,225], have been tested to support the best peri-implant tissue healing.
- Finally, only a few articles were found combining implants and drugs. Pilot studies have been performed to test topical use of implant surface treatments with melatonin coating or vitamin D [226,227] and the efficacy of mouth rinses for prevention of peri-implant mucositis and peri-implantitis, and more recently, the impact of hyperbaric oxygen on tissue healing was analyzed [228]. From a systemic point of view, vaccines have been developed seeking to prevent alveolar bone loss in peri-implantitis [229,230].
- Contribution of peri-implantitis studies in dog models to implantology: A specific strength of this model is the ability to perform periodontitis and peri-implantitis protocols, the dog being the large animal model most widely used in periodontitis studies [231]. A new line of research is the characterization of the peri-implantitis microbiota and changes therein during and after ligature placement, as well as after treatment [232,233,234,235]. Silk and cotton ligatures have been extensively used to initiate plaque formation and therefore an inflammatory process in gingival tissues [236]. New protocols have been developed to accelerate or exacerbate the inflammatory process [237]. For their flexibility and ease of handling, stainless steel ligatures have been proposed to replace soft ligatures which can be tricky to place and retain on the implantation site in the long term. Immediate induction of peri-implantitis has shown similar results to conventional methods with a shorter 6-month protocol [238].
- A better understanding has been obtained of implants’ susceptibility to bacterial contamination depending on the surface condition or composition [239] and characteristics [240,241,242] and tools have been developed to treat them mechanically (Ti-Brush [243], ER:YAG laser [243]) or with drugs (antimicrobials [244], chlorexidine [245], mouth rinse [246], or even plasma [247]) and to reconstruct bone tissue lost [248].

**Summary****:** A natural tendency to develop periodontal disease and the ease of reproducing peri-implantitis make the dog a strong model for dental implant research. Nonetheless, animal care regulations and husbandry requirements (exercise, need for environmental enrichment) [249] limit the use of dog models to research for which other animal models cannot be used (e.g., large models of peri-implantitis, microbiological studies) (Figure 5, Table A3). Interestingly, the emergence of new evidence demonstrating the similarity of dogs and humans in rare diseases [250] widens the field of application of this model, building a dual model with advantages of both large models (clinically relevant) and small models (genetic disorders).

**Table A3:** Overall appeal weighted by animal model. Rankings for each criterion from 1 to 6.

Models	Cost	Housing/Husbandry Requirement	Biological Interest	N per Animal	Ethical Issues	Protocol Duration	Surgical Relevance	Implant Model	Total
Mice	1	1	1	5	1	1	5	6	21
Rats	2	1	4	4	2	2	4	6	25
Rabbits	3	3	6	3	3	3	3	3	27
Pigs	4	4	4	1	4	5	2	1	25
Canines	5	5	3	2	5	4	2	1	27
Non-human primates	6	6	2	2	6	6	1	1	34
In vitro/in silico/biomaterials	1	1	-	-	1	1	7	7	/

### Appendix A.3. Research in Pig Models

Usually, 2- to 3-year-old animals [250] are used for implant insertion 3.5 months after tooth extraction and analysis 8.5 weeks after implantation, if a second stage is needed (Figure 2). Due to the substantial bone thickness and the presence of numerous adequate sites in the oral region, tibias have only been used in a few studies. Similarly, several protocols were developed in the skull (frontal bone or calvaria) but have now mainly been transposed to oral bone. The large amount of bone in the maxilla and mandible allows the use of several implants in a single protocol, and the performance of implant surgery in bone with more appropriate properties.

#### Appendix A.3.1. Oral Bone Models

Due to substantial oral bone thickness and the presence of other more appropriate areas, tibias have only been used in a few studies (Table A2). On the other hand, they allow researchers to (i) insert multiple implants in the same bone (up to 6 per tibia [251,252]), given their large surface area, and (ii) use a split design with both tibias, reducing the number of animals required.

Implant surgery can be performed following human protocols and using human-sized implants (mean of 4 mm in diameter and 11 mm in length). Therefore, primary stability, implant stability quotient, and removal torque can be measured in the same way as in clinical practice [252]. This model can also be used to test the viability and mechanical properties of new implants [253] or compare different models [251,254].

#### Appendix A.3.2. Skull Bone Models

Reports have been found of several studies the skull (frontal [255] or in calvaria [256]). These models allow implant osseointegration and healing without mastication constraints or tongue movement and are suitable for multiple-step protocols [257] or surgery with large instruments. This area also permits the implantation of up to 12 devices in the same animal. Nonetheless, the length of the implant has to be carefully selected to avoid brain damage. In terms of protocols, the scope for research is similar to that for the mandible: surface conditioning [258,259,260,261], implant composition [262], implant macro-design [263] and abutment [264], pull-out strength [265], and bone augmentation with [256] or without grafts [266], but also diseases (e.g., diabetes [267]).

#### Appendix A.3.3. Oral Bone Models

Most pig oral bone models use either the maxilla or mandible for implantology research. Not surprisingly, the maxillary sinus has been poorly studied due to its position (distal to the last molar) making it difficult to access. The maxilla and mandible can be used in the same protocol, often with teeth extractions in all four quadrants to maximize the number of implants [268]. In systemic disease processes, it seems that the pig model is poorly suited and only used for obesity/metabolic syndrome and/or diabetes [269,270,271]. Peri-implantitis also appears to have been poorly investigated [272].

A range of surgical techniques can be performed, such as (i) site preparation with different osteotomies [273], (ii) flap procedures [274,275], or (iii) bone surgery with osteodistraction [276], creation of bone defects [277], and bone grafting [271,278,279]. Biomaterials such as scaffold [280] and disc-shaped matrices for vertical bone regeneration [254,280] have been tested in pigs prior to use in human surgery. Biological coating functionalization with chondroitin sulfate and sulfated hyaluronan with collagen molecules [281,282,283] is often tested on this model, avoiding the process of miniaturization of a new model to be put on the market.

In oral bones, implants are almost always placed in a socket or healed site after tooth extraction [284]. There are numerous protocols for healing time (ranging from 9 weeks [285] to 8 months [273]), with a mean time of 3–4 months after tooth extraction. Notably, 3 to 4 implants can be anchored per quadrant [284] with human or human-like implants 4 mm in diameter and 11 mm in length [286].

As for the tibia, the oral model may be useful for histological analyses such as cytotoxicity and viability of new compositions [287,288,289,290] but also for assessments on 1:1 scale models of mechanical properties [47,268,291], stress distribution [287,288], and the effects of different implant and abutment shapes [264,284,292,293,294,295]. Thanks to pigs having similar bone properties to those of humans, resistance to insertion [273] or removal torque can be measured during [296] or after healing [268]. It is possible to perform classical analysis such as measurement of bone area [273], marginal bone level [273], crestal bone loss [275],^,^ resonance frequency [273], or implant stability quotient [297]. However, the main reason for selecting this site is almost always its suitability for bone-to-implant contact analysis [273,281,282,286,289,290,294]. This model is also used to assess the accuracy of new techniques, such as ultrasound imaging [298], comparison of intra-oral and cone beam computed tomography [299,300], magnetic resonance imaging [301], and resonance frequency analysis [302]. In addition, it can be used to investigate not only bone but also soft tissues [295] as in immunohistochemical analysis of blood vessels in peri-implant mucosa [274], cell quantification, and fiber orientation [292], or pangenomic gene-expression analysis [293] of implant tissues.

**Summary****:** Supported by a strong history of use, pig oral bone models are suitable for pre-clinical studies, allowing the testing of different implant surface properties or surgical procedures under physiological conditions. The benefits of minipigs in terms of costs and ease of housing make them the model of choice, overcoming the limitations of other large animal models (Figure 7).

### Appendix A.4. Research in Rabbit Models

Experiments are usually shorter than those in large animal models: (i) as previously described, bone maturity occurs at around 6 months of age, and hence, protocols use rabbits from 6 to 9 months of age, (ii) surgery is mainly performed in long bones, and hence, no healing time is needed, unlike protocols involving tooth extraction, and (iii) bone healing is faster. Protocols can stop 2 to 4 weeks after implantation for bone-to-implant contact analysis (Figure 2).

#### Appendix A.4.1. Long Bone Models

The great majority of studies in rabbits correspond to long bone experiments (69/86), with 50/69 on the tibia and 19/69 on the femur (Table A2).

- Protocols using the tibia: The anatomy and histology of rabbit tibia are well known, the rabbit being used for the first attempt to develop an animal model of osteomyelitis [303] for bone fracture analysis [304]. Numerous research protocols have been developed on the tibia, as the relatively good volume accessible has allowed analysis of as many as 112 implants in 28 rabbits in one study [305]. In this area, 3- to 4-mm diameter implants can be used with lengths of up to 7 mm [306]. Rabbit tibia has been widely used to analyze the osseointegration of zirconia implants [307], titanium–zirconium implants [308], implants coated with calcium carbonate [309], and implants with surface modifications [306,310,311].Bilateral procedures are generally described including (i) two implants per animal with one implant in each tibia [311]; (ii) four implants per animal with two implants in each [312] or (iii) six implants per animal with three implants per tibia [313,314]. The metaphysis and diaphysis of the bone can be used. Thanks to fast healing, osseointegration can be analyzed 1 month after implantation [312]. The tibia has also been used for drilling studies seeking to improve implant stability [315] with drilling speed [316] or drill diameter and implant torque [312] analysis. The large volume of the tibia and ease of surgery have allowed this bone to be used for the creation of peri-implant defects [317] and the use of a bone substitute model [318] and spacers [319], as well as for pathophysiological purposes, mainly for reduced bone models (osteopenic or osteoporotic conditions) [320,321,322,323]. Environmental parameters have been investigated in contexts such as a high-fat diet [324] and irradiation [325].
- Protocols using the femur: the femoral bone has been chosen by researchers for many reasons: Easy access and the small amount of soft tissue [326]. Rabbit long bones are composed of 70%–80% compact bone [68], allowing good implant stability.
- The femur is thicker than the tibia and the medullary space is large [326], allowing multiple implant fixations [63]. Experiments can also be performed on both sides of the knee (distal part of the femur and proximal part of the tibia) [327]. The disadvantages of this model are related to the general differences between humans and rabbits as mentioned above. In particular, rabbit long bones show a distinctive physiological variability of the bone architecture with a longitudinal vascular pattern [26]. Another point to consider is the age of the animal. Indeed, due to endosteal bone remodeling, the bone shows cortical thinning and an increase in bone marrow volume by as much as 24% with age [328]. It has also been reported that rabbit bone marrow contains a significant proportion of adipose tissue [5], a characteristic not present in the oral cavity in humans, and this reduces the usefulness of the model.

### Appendix A.4.2. Skull Bone Models

For the calvaria, only one study has been found. It sought to analyze the influence of nonsteroidal anti-inflammatory drugs on osseointegration of dental implants in the calvaria [329]. The model found no significant differences in the use of this type of drug and only one rabbit out of the 19 used died in the postoperative period.

### Appendix A.4.3. Oral Bone Models

The rabbit skull is mainly composed of spongy bone and contains wide spaces [69]. The mandible is formed by two symmetrical bones joined by a fibrous or fibrocartilaginous symphysis. As for humans, two parts can be described: (i) the horizontal part, which houses the teeth, and (ii) the vertical posterior part, called the ramus. The maxilla is also formed by two bones fused on the sagittal line. The dental formula of the rabbit is: two pairs of incisors on the maxilla and one on the mandible; no canines; three pairs of premolars on the maxilla and two on the mandible; and two to three pairs of molars on the maxilla and three on the mandible. The total number of teeth ranges from 26 to 28 [69]. Rabbit incisors grow continuously [330], which is of great interest for longitudinal mineralization studies [331].

As previously described, oral bone, in addition to the obvious difficulty of access due to the small mouth opening, has a poor cortical/spongy bone ratio with mainly spongy bone and wide spaces. There is a paucity of literature in this area and the models seem to be avoided by researchers (Table A1). Out of the 86 studies, 12 were performed in oral bone: 3 in the maxilla, 1 for sinus augmentation, and 8 in the mandible (Table A2).

- Studies in the maxilla: Studies in the maxilla are mainly used for related sinus augmentation therapies. Medications such as anti-inflammatory drugs and painkillers can be tested for postoperative pain [332]. Newly formed bone height is measurable following sinus floor elevation using a blood clot [333] or for pre-clinical testing of new bone substitute [334], giving an idea of how such materials are accepted in in vivo models. The poor bone quality of the maxillary sinus is also exploited for studying the impact of innovative surface properties in poor quality bone [335].
- Studies in the mandible: Procedures are short, immediate extraction/implantation protocols being the most common. The healing period after implantation is at least 3 weeks [336] and up to 3 months [337]. The incisor area provides a great volume for osseointegration in immediate extraction/implantation protocols with 3-mm diameter and 12-mm length implants [338]. Except for the study by Schiegnitz et al. using 9-month-old New Zealand rabbits (4–5 kg) [336], the age of rabbits is generally not specified accurately; rather, it is reported as “adult age” which corresponds to 2.5 to 6 Kg. Only one study found was performed in younger rabbits (4 months old), these having been exposed to fluoride since 2 months of age [337]. Studies in the rabbit mandible have been used to assess osseointegration of implants with different surface properties [339] or positions [336,340], and the systemic effect of exposure to molecules like fluoride [337] or the effect of thyroid hormone production [65]. It should be noted that one proof of concept study for a peri-implantitis model was conducted on the first mandible anterior tooth with silk ligatures in 2015 (reported only in Chinese, except for the abstract [341]). In the mandible, an extra-oral approach by opening a flap from the skin to the mandible angle has been used for vertical bone growth, making it possible to extend the scope of already known materials [342]. The great volume available in this area allows the use of human-sized implants, with a length of 8 mm and a diameter of 4.1 mm.

**Summary****:** Rabbit models have an extensive history of use and well-developed protocols in studies in orthopedics and more recently periodontal diseases. The weak bone quality around the oral cavity, however, limits the use of long bone in this model for implantology, biocompatibility, or osteoinduction [6].

Nonetheless, rabbit models allow analysis of numerous implants per animal, thereby reducing the total number of animals needed per protocol (Table A1). Their application remains limited due to the small number of genetic models.

### Appendix A.5. Research in Rat Models

Protocol durations depend on the site, with substantial differences between studies. The average age of animals is around 2 to 3 months for the first surgery. For long bone procedures, 2 to 6 weeks is needed before assessing osseointegration. In the case of implant placement at a healed extraction site, 1.5 months of healing is generally allowed after extraction and another month for implant osseointegration (Figure 2). In the maxilla, protocols are shortened, with implantation performed immediately after extraction.

#### Appendix A.5.1. Long Bone Models

- Studies in the tibia: The rat tibia is suitable for bone implantation due to the ease of access and relatively good volume. Notably, 32 out of the 68 studies found have been performed with this bone. The medial tibial plate of the bone is commonly used as it is flat and can receive implants [343]. Depending on the prototype used, the number of tests and their size differ. At least one implant per tibia can be tested with a nearly human-size implant (2.0 mm in diameter and 4- to 5-mm in length [83,344,345,346]). On this kind of model, bi-cortical anchorage can be achieved. For multi-implant protocols, a diameter of 1.5 mm and length of 2.5 mm are more appropriate [343,347].
- Research using the rat tibia model has commonly investigated the effects of the implant surface on osseointegration [343,344], but a new trend has emerged, with growing numbers of studies in the areas of the drug delivery and/or physiopathology: effects on osseointegration of different doses of drugs in rats that are healthy [345] or have certain diseases, e.g., diabetes [348,349,350,351,352,353,354], or in peri-implant bone defects [346]. Nevertheless, the framework of choice is implantation osseointegration in poor quality bone with drug treatments [355,356]. Research into bisphosphonates [347,355,357,358,359] or agonists like selective estrogen receptor modulators [347,360] is typically conducted in the rat as it is an excellent model of osteoporosis. Other diseases, such as arthritis [361] or Crohn’s disease [362] and the effects of severe dietary magnesium deficiency on systemic bone density, have been investigated [363]. Finally, the rat tibia can also be used for mechanical testing, including in dynamic loading models [364,365], as well as for exploring the effects of pulsed ultrasound [366] or laser therapy [367].
- Studies in the femur: Only a few studies have been conducted in femoral bone mainly due to (i) the short length of the bone and (ii) the amount of muscle and tissue surrounding it. Nonetheless, titanium mesh [368] and implants have been placed for surface testing [369] in diabetic rats [370,371,372] or in combination with dietary supplements [373] or cell therapy [374].

#### Appendix A.5.2. Oral Bone Models

Research in implantology tends to focus on oral bone for the sake of model legitimacy. In small animals, like rats, protocols have to be adapted to be suitable. Various strategies for the maxilla and mandible have been developed in the rat and illustrate some ways protocols can be adapted. Tooth extraction and subsequent dental implant placement is the best proxy procedure in terms of “human-like protocols”.

- Studies in the maxilla: Numerous studies have assessed the validity of the maxillary molar site, but with no established guidelines and considerable heterogeneity between protocols. For this procedure, the implants or “mini-screws” measure approximately 1 mm in width and 2 mm in length, though some authors prefer longer and wider implants for good primary stability despite the increase in the risk of sinus perforation [375]. In any case, maxillary molars seem to be an adequate place for implant–prototype anchorage immediately after extraction [376] or with a delay [375,377]. Another possibility is to use the maxillary diastema, mesial to the molars. A recent study has successfully shown a model of peri-implantitis in this area [378], but the validity of this model has yet to be demonstrated [379]. The maxilla is also used for classic implant surface comparisons [376], mechanical testing [380], and analysis of pathophysiological processes [381,382,383,384,385].
- Studies in the mandible: Beyond the maxilla, two studies have been found that used the mandibular region: one protocol used the posterior part of the mandible in the ramus through an extra-oral approach [386]; and in the other, the implant took the place of the first mandibular molar after extraction and healing for a month [387]. Both protocols led to significant osseointegration, but the latter seems to be more physiologically relevant as it replaces a previous tooth in alveolar bone.
- Contribution of peri-implantitis studies in rat models to implantology: In periodontology, in humans, as in many other animals, chronic inflammation needs to be produced at the sulci of the tooth (with silk ligatures and/or microbial gavage) to produce periodontal destruction [388]. In the same way, peri-implantitis can be induced with a mixed bacterial infection (*Streptococcus oralis* and *Aggregatibacter actinomycetemcomitans*) [378].

**Summary****:** The rat offers numerous advantages for implantology studies: it is a well-known model for systemic variation (diabetes, hormones), and therefore, for osseointegration in models of poor bone quality (Table A1). On the other hand, opportunities for genetic studies remain limited compared to those with mouse models.

### Appendix A.6. Research in Mouse Models

It seems that 8 weeks is the minimum age of mice for implantation. For protocols in oral bone, 4 to 8 weeks are needed for alveolar healing and 3–4 weeks for implant osseointegration. This is the same length of time as for osseointegration in the tibia (Figure 2).

#### Appendix A.6.1. Back of the Mouse

Only one study was found using the back of mice. It sought to analyze the potential bacterial infection originating from the implant itself during surgery. This model was used as it offered a closed environment with no potential interaction, unlike in the case of the mouth [389].

#### Appendix A.6.2. Long Bone Models

- Studies in the tibia: with only two studies found from this decade, it seems that the shin has fallen into disuse. One was considered a mouse study but the animal species used was actually *Rattus norvegicus*, that is, the article had been erroneously classified [390], and the other was conducted to analyze peri-implant bone density in senescence-accelerated mice, but the choice of the shin over femur was not explained in detail [391].
- Studies in the femur: The femur is more commonly used, but nonetheless only a few studies were found. The most common topic is the evaluation of implant osseointegration associated with disease. Diabetes is the most studied disease in association, for example, with drug therapies (1α,25-Dihydroxyvitamin D3 [392]; transcription factors [HIF-1α] [393]). Innovative genetic technologies for lentiviral vector transfection are also useful for testing new treatments [394], or a specific molecular pathway [395]. For this bone, the common prototype is an implant-like model, mostly with a pin-shaped implant 1 mm in diameter and 2- to 3-mm in length [392,394]. Titanium discs are also used if the aim is to test the biocompatibility of the material [395].

#### Appendix A.6.3. Oral Bone Models

- Studies in the maxilla: No studies were found on the mandible due to the difficulty of access and mechanical difficulties for manufacturing miniature implants [4]. The maxilla is a relatively recent model, having been developed during the last 10 years [396]. It has become the most common model used in mice (Table A2). Nonetheless, due to the recent description of this model, research is focused on the development of the model itself more than on its implementation. The first model in the dental area, reported in 2014, used “retopins” (0.6-mm diameter cut to a 2-mm length, NTI Kahla GmbH, Germany) positioned in the mesial part of the first maxillary molar. This model demonstrated that osseointegration in oral bone cannot be compared to long bone studies [87,397], but it has recently been shown to be a suitable tool for the assessment of biological events associated with the osseointegration process [398]. This protocol has also been adapted for analysis of (i) immediate post-extraction implant placement [399,400,401] and (ii) the involvement of different pathways [397,402]. This site can also be used to test new implant compositions, such as a bio-implant [403].
- Contribution of peri-implantitis studies in mouse models to implantology: In order to investigate how to manage infectious conditions, there is also a need to identify a new model of peri-implantitis in the maxilla. Five different studies have proposed different peri-implantitis models:
  o The first one was also the first study to anchor an implant in the oral cavity [396]. The pin-shaped implant was placed in the medial line of the hard palate and peri-implantitis was induced with a special diet enriched with sugar and flavorings.
  o Pirih et al. developed two new peri-implantitis models with a 1-mm length/0.5-mm diameter implant placed in the second and third maxillary molar area: (1) using silk ligatures placed apically to the implant head [404] and (2) using lipopolysaccharide injections on the implant surface [405].
  o Another silk model validated the maxillary molars as a potential site for peri-implantitis [406]. This model was later used for the comparison of peri-implantitis and periodontitis progression [407].
  o Peri-implantitis was also obtained in a recent study of oral infection with *Porphyromonas gingivalis* [408]. Since the model has been well established, some applications have emerged, namely, analysis of the impact of different implant surfaces on peri-implantitis [409] or inflammation pathways [410].

**Summary****:** Mice seem to be a promising model for genetic research thanks to the ability to perform knock-out studies and the availability of models of human disease. New approaches are emerging, such as peri-implantitis protocols, extending the range of possibilities in this model (Table A1).

On the other hand, mice have obvious limitations typical of small animal models. Implant adaptation in the oral cavity is a limitation both from a surgical and industrial point of view (Figure 4). This disadvantage should be seen as a challenge and may be addressed thanks to technological advances.
